# Angina After Percutaneous Coronary Intervention: Patient and Procedural Predictors

**DOI:** 10.1161/CIRCINTERVENTIONS.122.012511

**Published:** 2023-03-28

**Authors:** Damien Collison, Samuel Copt, Takuya Mizukami, Carlos Collet, Ruth McLaren, Matthaios Didagelos, Muhammad Aetesam-ur-Rahman, Peter McCartney, Thomas J. Ford, Mitchell Lindsay, Aadil Shaukat, Paul Rocchiccioli, Richard Brogan, Stuart Watkins, Margaret McEntegart, Richard Good, Keith Robertson, Patrick O’Boyle, Andrew Davie, Adnan Khan, Stuart Hood, Hany Eteiba, Colin Berry, Keith G. Oldroyd

**Affiliations:** West of Scotland Regional Heart & Lung Centre, Golden Jubilee National Hospital, Glasgow, United Kingdom (D.C., R.M., M.D., M.A.R., P.M., M.L., A.S., P.R., R.B., S.W., M.M., R.G., K.R., P.O., A.D., A.K., S.H., H.E., C.B., K.G.O.).; Institute of Cardiovascular & Medical Sciences, University of Glasgow, United Kingdom (D.C., P.M., T.J.F., S.W., M.M., R.G., H.E., C.B., K.G.O.).; University of Geneva, Switzerland (S.C.).; Cardiovascular Center Aalst, OLV Clinic, Belgium (T.M., C.C.).

**Keywords:** angina pectoris, coronary artery disease, myocardial infarction, myocardium, percutaneous coronary intervention, quality of life

## Abstract

Twenty percent to 40% of patients are affected by angina after percutaneous coronary intervention (PCI), which is associated with anxiety, depression, impaired physical function, and reduced quality of life. Understanding patient and procedural factors associated with post-PCI angina may inform alternative approaches to treatment.

**Methods:**

Two hundred thirty patients undergoing PCI completed the Seattle Angina Questionnaire (SAQ-7) and European quality of life–5 dimension–5 level (EQ-5D-5L) questionnaires at baseline and 3 months post-PCI. Patients received blinded intracoronary physiology assessments before and after stenting. A post hoc analysis was performed to compare clinical and procedural characteristics among patients with and without post-PCI angina (defined by follow-up SAQ-angina frequency score <100).

**Results:**

Eighty-eight of 230 patients (38.3%) reported angina 3 months post-PCI and had a higher incidence of active smoking, atrial fibrillation, and history of previous myocardial infarction or PCI. Compared with patients with no angina at follow-up, they had lower baseline SAQ summary scores (69.48±24.12 versus 50.20±22.59, *P*<0.001) and EQ-5D-5L health index scores (0.84±0.15 versus 0.69±0.22, *P*<0.001). Pre-PCI fractional flow reserve (FFR) was lower among patients who had no post-PCI angina (0.56±0.15 versus 0.62±0.13, *P*=0.003). Percentage change in FFR after PCI had a moderate correlation with angina frequency score at follow-up (*r*=0.36, *P*<0.0001). Patients with post-PCI angina had less improvement in FFR (43.1±33.5% versus 67.0±50.7%, *P*<0.001). There were no between-group differences in post-PCI FFR, coronary flow reserve, or corrected index of microcirculatory resistance. Patients with post-PCI angina had lower SAQ-summary scores (64.01±22 versus 95.16±8.72, *P*≤0.001) and EQ-5D-5L index scores (0.69±0.26 versus 0.91±0.17, *P*≤0.001) at follow-up.

**Conclusions:**

Larger improvements in FFR following PCI were associated with less angina and better quality of life at follow-up. In patients with stable symptoms, intracoronary physiology assessment can inform expectations of angina relief and quality of life improvement after stenting and thereby help to determine the appropriateness of PCI.

**Registration:**

URL: https://www.clinicaltrials.gov; Unique identifier: NCT03259815.

**What is Known**

- Angina after percutaneous coronary intervention (PCI) is associated with long-term anxiety, depression, and impairment of both physical function and quality of life.
- Persistence or recurrence of angina after PCI may affect 20% to 40% of patients during short-to-medium-term follow-up.

**What the Study Adds**

- Patients with post-PCI angina reported more frequent angina and poorer quality of life at baseline yet had physiologically less severe disease and, accordingly, tended to achieve less improvement in coronary physiology metrics than those who were free from angina post-PCI.
- The degree of improvement in patient-reported outcome measures following PCI is not associated with post-PCI coronary physiology values. It relates instead to the pre-PCI value and the change from baseline. Lower pre-PCI values and larger increases in fractional flow reserve were associated with higher patient-reported outcome measures scores at follow-up.
- Intracoronary physiology assessment can inform expectations of angina relief and quality of life improvement after stenting and thereby help to determine the appropriateness of PCI intended to alleviate symptoms.

**See Editorial by Smilowitz and Seto**

Angina after percutaneous coronary intervention (PCI) is associated with long-term anxiety, depression, impaired physical function, and quality of life.^1^ PCI achieves greater reductions in myocardial ischemia than optimal medical therapy alone.^2^ The greater the degree of ischemia in a myocardial territory, the greater the improvement in symptoms following PCI.^3^ Patients with moderate or severe ischemia randomized to an initial invasive strategy in the ISCHEMIA trial (International Study of Comparative Health Effectiveness With Medical and Invasive Approaches) had greater improvement in angina-related health status than those assigned to the conservative strategy, and larger differences were observed in patients who actually had anginal symptoms at baseline.^4^ Nevertheless, persistence or recurrence of angina after PCI is well-recognized and may affect 20% to 40% of patients during short-to-medium-term follow-up.^5,6^ Total health care costs in the first year after an index PCI can be up to 1.8× greater for patients with angina or chest pain after stenting with cost differentials continuing out to 36 months post-PCI.^7^ Understanding patient factors associated with post-PCI angina may support different approaches to revascularization.^8^ Conflicting data exist regarding the association between invasive coronary measurements and patient-reported outcome measures at follow-up.^9–11^ TARGET-FFR (Trial of Angiography Versus Pressure-Ratio-Guided Enhancement Techniques - Fractional Flow Reserve) was a randomized controlled trial designed to assess the efficacy of a post-PCI physiology-guided incremental optimization strategy versus standard angiographic guidance.^12^ In this analysis, the incidence and associates of angina at 3 months post-PCI were examined.

## Methods

The data that support the findings of this study are available from the corresponding author upon reasonable request. The rationale, study design, and primary outcome of the TARGET-FFR controlled trial have been published previously.^12,13^ In brief, 260 patients undergoing standard-of-care PCI for either chronic or medically stabilized acute coronary syndromes provided informed consent to be randomized 1:1 to a control group or the experimental physiology-guided incremental optimization strategy. All patients completed questionnaires on anginal symptoms (Seattle Angina Questionnaire [SAQ-7]) and health status (European quality of life–5 dimension–5 level [EQ-5D-5L]) at baseline and were contacted to repeat this assessment 3 months after their procedure. The questionnaires were administered by telephone or mail by a research nurse blinded to the randomized group allocation and the physiology results. A summary of the study protocol and additional detail on the patient questionnaires is provided in methods in the Supplemental Material. The study was approved by an institutional review committee, and patients gave informed consent.

### Definition of Angina

The presence of angina post-PCI was defined by a patient-reported follow-up SAQ-angina frequency (SAQ-AF) score of <100. Patients with a follow-up of SAQ-AF score=100 were classified as having no angina.^4,8^ Prior to PCI, in addition to patient-reported SAQ scores, anginal symptoms were also assessed and adjudicated by a physician with a Canadian Cardiovascular Society (CCS) score of class I or above defining the presence of angina at baseline. This definition was used for subgroup analyses of patients with anginal symptoms at baseline.

### Clinical Outcomes

Clinical outcomes at a median of 3 years post-PCI were assessed by electronic health record linkage. The primary clinical outcome was target vessel failure, a composite end point comprising cardiovascular death, target vessel myocardial infarction, and target vessel revascularization.^14^

### Statistical Analysis

Continuous variables are presented as mean±SD and categorical data as counts and percentages. A 2-sample *t* test was used to compare patient-level characteristics with continuous variables. Categorical variables were compared using the χ^2^ test without continuity correction. Whenever appropriate, a Fisher exact test was used instead. Follow-up patient-reported outcome measures scores stratified by FFR tertiles were analyzed with an ANCOVA model on the parameter’s follow-up value adjusted for FFR tertiles and baseline value. Relationship between variables was assessed using Spearman correlation coefficient. Analyses were performed using SAS 9.4 and SPSS Statistics 28.

## Results

Of 260 participants, 230 (88.5%) provided follow-up SAQ-AF scores 3 months (median [interquartile range], 105 [31] days) post-PCI. For the purposes of this analysis, patients were stratified by the presence of post-PCI angina. Eighty-eight (38.3%) of 230 patients had post-PCI angina as determined by a follow-up SAQ-AF score <100. Expanded subgroup analyses with additional stratification by the presence of angina at baseline are included in the (Tables S1 through S5). Five of eighty-eight (5.7%) patients with post-PCI angina did not have angina (physician-adjudicated CCS class ≥1) at baseline. There were no periprocedural myocardial infarctions within this subgroup and mean post-PCI physiology values compared favorably to the other subgroups (Table S2).

### Baseline Demographics

Clinical characteristics at baseline are presented in Table 1. Patients with post-PCI angina had higher rates of previous myocardial infarction and PCI. The incidence of atrial fibrillation and current cigarette smoking were also higher in those with post-PCI angina. Patients with post-PCI angina had significantly higher CCS scores at baseline and were prescribed more antianginal drugs with greater utilization of oral nitrate tablets and more frequent use of reliever sublingual nitrate spray.

**Table 1.**
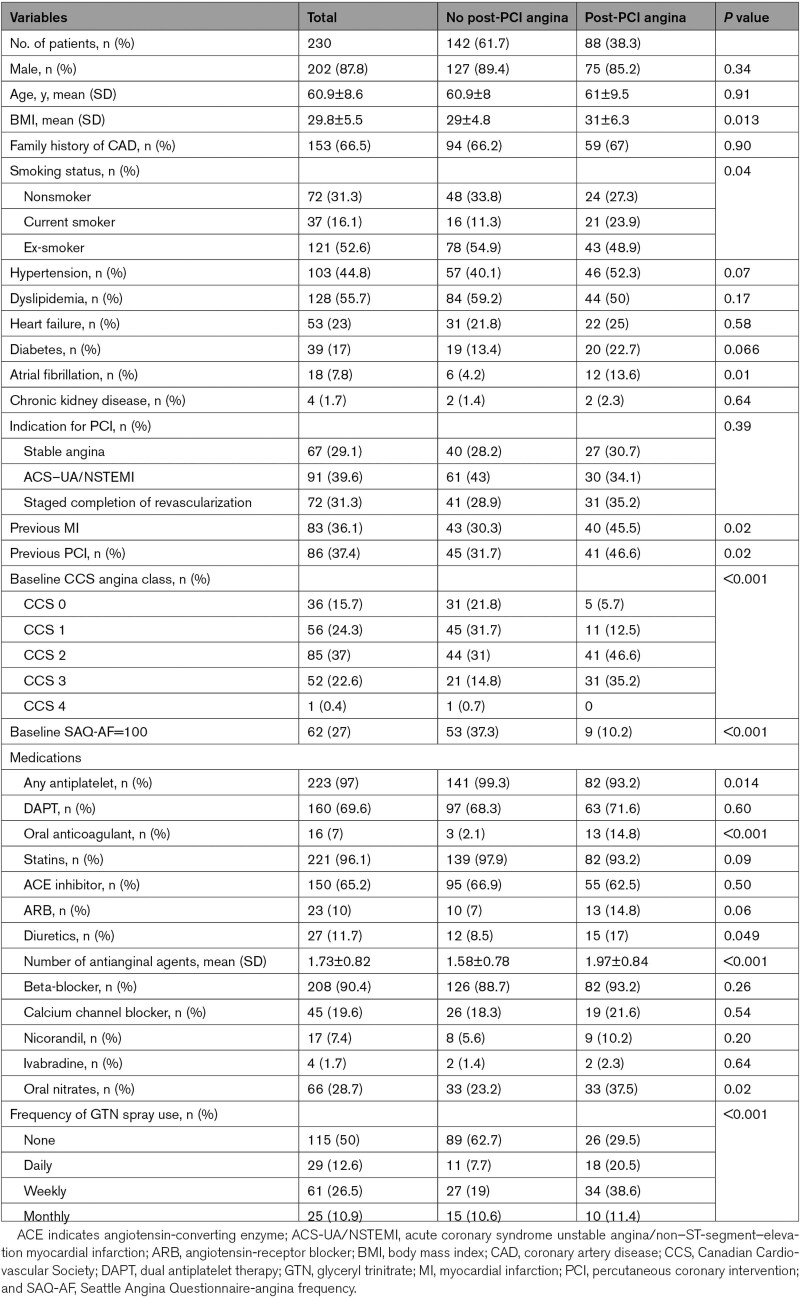
Baseline Clinical Characteristics Stratified by Presence of Angina 3 Months Post-PCI

### Procedural Outcomes

Procedural and coronary physiology characteristics are presented in Table 2. There were no differences between groups in the angiographic severity of stenoses or procedural characteristics such as lesion preparation, stent length, postdilation, and use of intracoronary imaging. Patients who were angina-free at follow-up had physiologically more severe lesions prior to PCI and achieved significantly larger improvements in hyperemic and nonhyperemic pressure ratios after stenting. There were no between-group differences in either post-PCI physiology metrics or in the proportion of patients who received additional intervention through the study’s post-PCI physiology-guided optimization protocol.

**Table 2.**
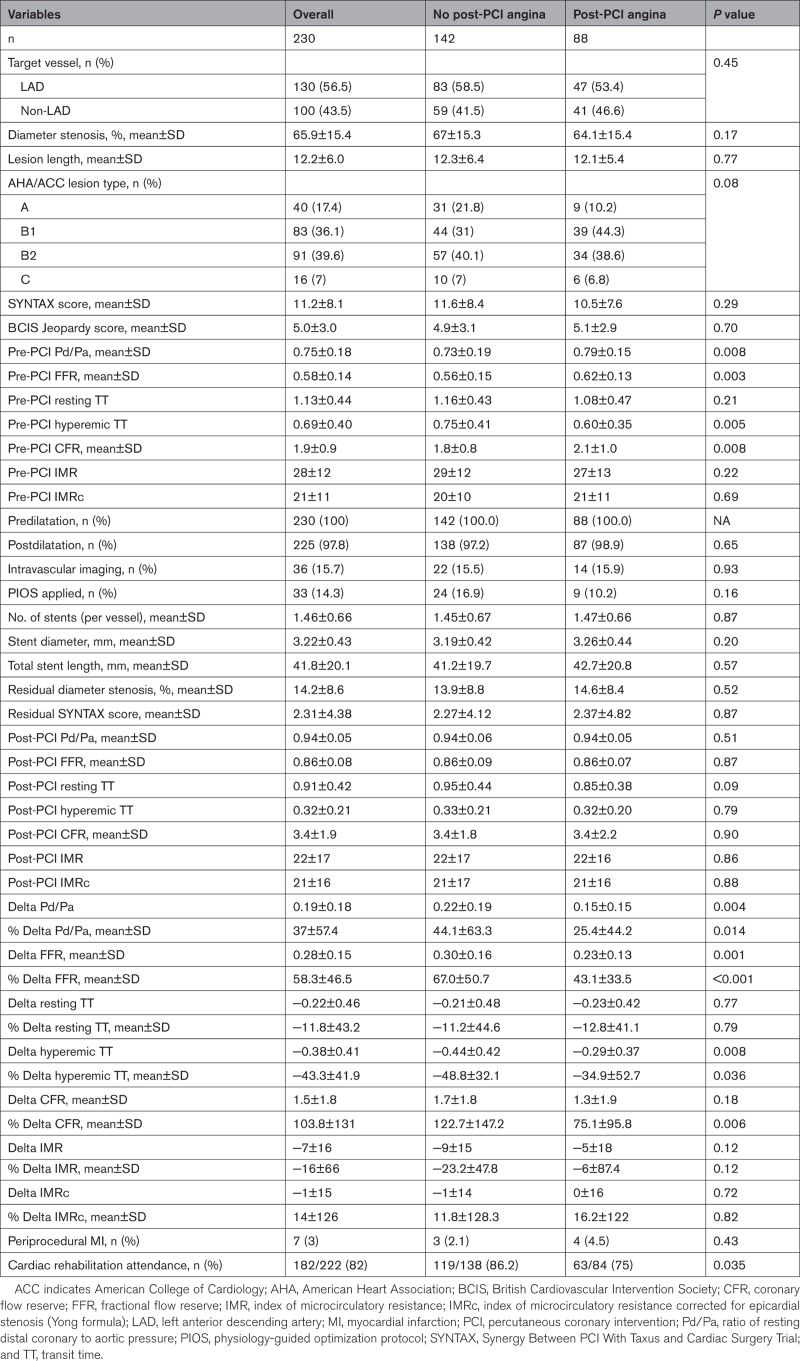
Procedural and Coronary Physiology Characteristics Stratified by Presence of Angina 3 Months Post-PCI

### Patient-Reported Outcome Measures

Patients with post-PCI angina had significantly lower SAQ scores, both at baseline and follow-up, compared with those free from angina (Table S6). Additionally, in those patients reporting any angina after PCI, the mean change in the EQ-5D-5L Weighted Health Index was effectively zero (−0.001 units), indicating they felt their quality of life had not improved after stenting (Table S7). Among patients who had angina at baseline (CCS class I and above), FFR and coronary flow reserve (CFR) correlated with the absolute patient-reported outcome measures scores at follow-up. FFR had a moderate, positive correlation with follow-up SAQ-AF scores. CFR had a similar, albeit weaker, correlation. This reflected negative correlations with the pre-PCI SAQ-AF score rather than positive correlations with post-PCI values (Tables S8 through S11). Pre-PCI and change in FFR (delta FFR) both also had significant, albeit somewhat weaker, correlations with the other SAQ domains and the EQ-5D-5L weighted health index score (Tables S8 and S9). The magnitude of change in pressure ratios was predicated by the pre- rather than post-PCI value and patients reporting angina post-PCI tended to have achieved smaller changes in FFR following PCI (Figure; Figure S1). There was no correlation between index of microcirculatory resistance (corrected for epicardial stenosis) and patient-reported outcome measures (Tables S12 and S13).

**Figure.**
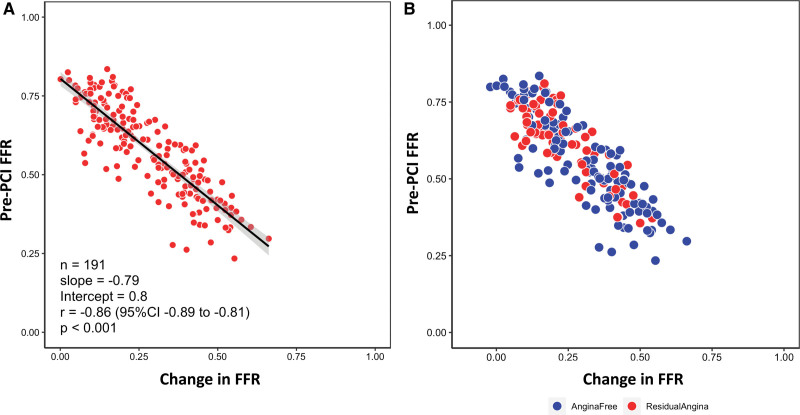
**Correlation between pre-percutaneous coronary intervention (PCI) fractional flow reserve (FFR) and change in FFR values. A**, The strong negative correlation between these variables−lower pre-PCI FFR values correlate with larger changes in FFR after PCI. **B**, Additional stratification by the presence of angina 3 mo post-PCI (red dots).

### Predictors of Post-PCI Angina

Univariate and multivariate analysis of predictors of post-PCI angina are presented in Table 3. Smaller changes in FFR predicted the presence of post-PCI angina.

**Table 3.**
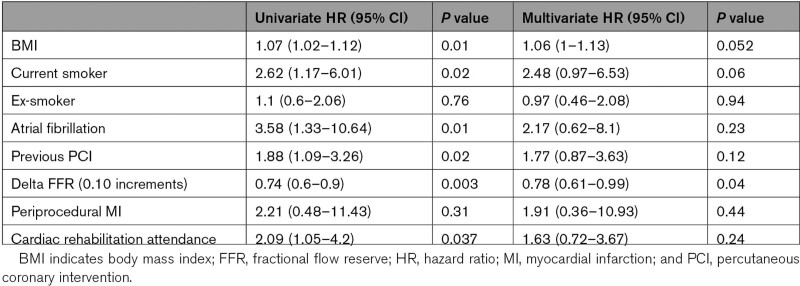
Predictors of Post-PCI Angina

### Clinical Outcomes

The rate of target vessel failure at a median (interquartile range) follow-up of 3 (0.9) years was 1.7% (4/230) with no significant difference between groups (no angina, 0.7% versus post-PCI angina, 3.4%; *P*=0.16), however, the study was not powered to detect a difference in clinical outcomes.

## Discussion

One in 3 patients in the TARGET-FFR randomized trial reported angina 3 months after undergoing PCI which, while a substantial proportion, is not unprecedented. In the ABSORB IV trial, 39% (494/1265) of patients in the drug-eluting stent arm had physician-adjudicated angina or angina-equivalent symptoms at 1-year follow-up.^5^

As the impact of anginal symptoms is inherently subjective, we concluded that attempting to define a level of post-PCI angina that might be acceptable to patients or represent a clinically meaningful improvement would be arbitrary and ultimately futile. Improved but persistent symptoms may be considered a success by some patients yet be completely unacceptable to others, so we therefore, determined a complete absence of patient-reported angina after PCI to represent the gold standard. The SAQ-AF domain asks patients to report the frequency of “chest pain, chest tightness, or anginal attacks” over the preceding 4 weeks with a score <100 indicating at least one anginal episode within that period. Accordingly, we adopted a follow-up SAQ-AF score of <100 as the definition of post-PCI.

Approximately 6% of participants reporting angina after stenting did not have symptoms prior to their intervention which highlights the importance of ascertaining the indication for and appropriateness of PCI with patients prior to embarking upon the procedure, particularly for those who are asymptomatic.

Patients reporting post-PCI angina had a higher burden of cardiovascular risk factors, including cigarette smoking, prior myocardial infarction, and atrial fibrillation at baseline. The higher rates of oral anticoagulants and concomitant lower rate of antiplatelets prescribed at baseline among patients reporting post-PCI angina are likely just commensurate with the higher incidence of atrial fibrillation in this group. As there was no difference in the incidence of the heart failure or hypertension, the higher rate of diuretic therapy may relate to prescriptions for dyspnea thought to represent an angina-equivalent symptom.

Patients with post-PCI angina had more severe symptoms at baseline (higher incidence of both CCS class 2 and 3 angina and self-reported rates of daily and weekly angina) and were prescribed more antianginal agents with greater use of oral and sublingual nitrates than those who were angina-free postprocedure. Patients experiencing any angina post-PCI reported effectively no improvement in quality of life as assessed by the mean change in their EQ-5D-5L weighted health index score (Table S7) and had significantly lower SAQ quality of life scores compared with those who were angina free (Table S6). These findings are contrary to a previous report concluding that preprocedural angina frequency is the most important prognostic indicator of quality of life after PCI.^15^

### Procedural and Intracoronary Physiology Characteristics

There were no significant differences between groups in angiography-based parameters of coronary artery disease severity either pre- or post-PCI. Counterintuitively, patients with post-PCI angina had a higher burden of angina at baseline yet physiologically less severe lesions (significantly higher pre-PCI FFR and CFR values and faster hyperemic transit times) than those who were angina-free at follow-up. There were no differences in absolute post-PCI physiology values between groups, and among patients with angina at baseline, there was no correlation between post-PCI values and patient-reported outcome measures at follow-up. Coronary microvascular dysfunction has been proposed as a potential mechanism for persistent angina post-PCI.^6^ In this population of patients with obstructive epicardial coronary disease, there were no differences in mean post-PCI CFR or index of microcirculatory resistance values between those patients with or without post-PCI angina. Based on currently accepted thresholds, the mean post-PCI CFR and index of microcirculatory resistance corrected for epicardial stenosis values in the post-PCI angina group of 3.4 and 21, respectively, were not suggestive of coronary microvascular dysfunction. Furthermore, there was no correlation between corrected index of microcirculatory resistance and patient-reported outcome measures. It seems unlikely that these patients went on to develop de novo coronary microvascular dysfunction over the following 3 months, however, the study design did not include invasive or noninvasive assessments of microvascular function at this time point. Acetylcholine provocation testing was not performed, therefore, the incidence of coronary vasospasm is unknown.

A physiology-stratified analysis of the ORBITA trial (Objective Randomised Blinded Investigation With Optimal Medical Therapy of Angioplasty in Stable Angina) assessed paired SAQ and EQ-5D-5L data from 189 patients and found that pre-PCI FFR and the instantaneous wave-free ratio (iFR) did not predict the effect of placebo-controlled PCI on anginal symptoms or quality of life.^9^ A subsequent analysis of pooled data from the FAME (Fractional Flow Reserve Versus Angiography for Multivessel Evaluation) 1 and 2 trials reported that larger improvements in FFR with PCI were associated with an increased probability of improvement of at least 2 CCS classes at 1-month follow-up but did not find any correlation between pre-PCI FFR values and symptom improvement.^11^ Latterly, another pooled analysis of FAME 1 and 2 data found that lower pre-PCI FFR, higher delta FFR and higher percentage delta FFR were associated with significantly larger change from baseline EQ-5D index score at both 1 month and 1 year post-PCI.^10^

In the present analysis, among patients who had angina at baseline (CCS class I and above), pre-PCI, absolute and percentage delta FFR values had significant correlations with patient-reported outcome measures at follow-up. Post-PCI values had no correlation with angina or quality of life at follow-up indicating that, as demonstrated in the Figure and Figure S1, pre-PCI values drove the magnitude of the change. Larger change in FFR following PCI was associated with higher patient-reported outcome measures scores at follow-up. In keeping with these findings, significantly lower absolute and percentage change values for FFR were observed in the post-PCI angina group. After multivariate analysis, delta FFR was found to be an independent predictor of post-PCI angina. Patients with a higher symptom burden and lower quality of life scores at baseline are more likely to report post-PCI angina, particularly where PCI can only achieve a small improvement in physiology metrics, such as in those with diffuse patterns of coronary disease and borderline or gray-zone pre-PCI values. Accordingly, this analysis from the TARGET-FFR randomized trial supports the concept that intracoronary physiology assessment can inform expectations of angina relief and quality of life improvement after stenting and thereby help to determine the appropriateness of PCI intended to alleviate symptoms. Patients with physiologically severe lesions can expect a larger improvement in intracoronary pressure ratios following PCI which is associated with a higher likelihood of angina relief and improved quality of life.

### Limitations

TARGET-FFR was a single-center study with a relatively homogeneous PCI practice, including high rates of lesion predilatation and high-pressure stent postdilatation. A larger multicenter trial incorporating a wider range of PCI strategies and techniques may have had a different outcome. The study was not powered for clinical outcomes and the incidence of target vessel failure at a median follow-up of 3 years remained low.

### Conclusions

The magnitude of FFR improvement following PCI correlated with angina status and quality of life at follow-up and was an independent predictor of the presence of post-PCI angina. In patients with stable symptoms, intracoronary physiology assessment can aid prediction of angina relief and quality of life improvement after stenting and thereby help to determine the appropriateness of PCI.

## Article Information

### Sources of Funding

Endowment funds at the Golden Jubilee National Hospital (NHS Golden Jubilee), Glasgow, United Kingdom, with support from the British Heart Foundation (Research Excellence Award RE/18/6/34217).

### Disclosures

Dr Collison received consultancy fees from Abbott. Dr Mizukami received consultancy fees from Zeon Medical Inc; research grants from Boston Scientific; speaker fees from Abbott, Cathworks, Boston Scientific. Dr Collet received research grants from Biosensors, Coroventis Research, Medis Medical Imaging, Pie Medical Imaging, CathWorks, Boston Scientific, Siemens, HeartFlow, Abbott; consultancies HeartFlow, OpSens, Abbott, Philips Volcano. Dr Ford received consulting fees from BioExcel; honoraria from Abbott, Boehringer, Novartis. Dr Watkins received honoraria from Abbott, AstraZeneca, Biosensors, Boston Scientific, Daiichi Sankyo, GE Healthcare, ShockWave Medical. Dr Robertson received honoraria from AstraZeneca, Abbott. Dr O’Boyle received honoraria from AstraZeneca, Boston Scientific, Novartis. Dr McEntegart received consulting fees from Abbott, Boston Scientific, ShockWave Medical; honoraria from International Medical Device Solutions, Medtronic. Prof Berry received institutional grants/contracts from Abbott, AstraZeneca, Boehringer Ingelheim, GlaxoSmithKline, HeartFlow, Novartis, Siemens Healthcare; consulting fees from Abbott, AstraZeneca, Boehringer Ingelheim, GlaxoSmithKline, HeartFlow, Menarini, Novartis; honoraria from Abbott, AstraZeneca, Boehringer Ingelheim, GlaxoSmithKline, HeartFlow, Philips, Valo Health. Prof Oldroyd received honoraria from Abbott, Biosensors International, Boston Scientific; institutional research grant from Boston Scientific which supported the present manuscript; full-time employee of Biosensors International since May 2020. The other authors report no conflicts.

### Supplemental Material

Supplemental Methods

Figure S1

Tables S1–S13

## Supplementary Material


