# Supplementary material for: Angina After Percutaneous Coronary Intervention: Patient and Procedural Predictors
Source: Circ Cardiovasc Interv. 2023 Mar 28;16(4):e012511. doi: 10.1161/CIRCINTERVENTIONS.122.012511 (PMC10101135; doi:10.1161/CIRCINTERVENTIONS.122.012511)
Supplement: Supplementary file 1 [file hcv-16-e012511-s001.pdf]

# Angina after percutaneous coronary intervention: patient and procedural predictors

Collison et al

## Supplemental Material

- Supplemental Methods
- Figure S1
- Tables S1 – S13

**Supplemental Methods**

Patients in the TARGET-FFR trial received a comprehensive pre-PCI coronary physiology assessment including resting distal coronary to aortic pressure (Pd/Pa), fractional flow reserve (FFR), coronary flow reserve (CFR), the index of microcirculatory resistance (IMR) and a hyperemic pressure-wire pullback was encouraged (PressureWire X Guidewire, Abbott Laboratories, IL, USA and CoroFlow v3.0 software, Coroventis Research AB, Uppsala, Sweden). Randomization was performed prior to a mandatory, blinded post-PCI coronary physiology assessment as above. Results were only disclosed to operators for patients in the treatment arm who had a post-PCI FFR <0.90. The operator then followed the steps of the PIOS algorithm to optimize the final PCI result. A hyperemic trans-stent gradient (HTG) ≥0.05 was treated with additional stent post-dilation with a larger non-compliant balloon. A focal (abrupt) drop of ≥0.05 FFR units on pullback through an unstented segment represented a target for additional stenting. If the residual pressure gradient reflected diffuse atherosclerosis with no focal step-changes in pressure gradient, the result was accepted and no optimization attempted. Final coronary physiology results were not disclosed to patients.

*Patient-Reported Outcome Measures*

Seattle Angina Questionnaire (SAQ) scores range from 0 to 100, with higher scores indicating better health status. The EQ-5D-5L comprises 2 components: a descriptive profile and a single-index visual analog scale (VAS). The descriptive profile assesses 5 dimensions of general health (mobility, self-care, usual activities, pain/discomfort, and anxiety/depression) with a 5-level scale. Higher scores indicate more severe limitation within that dimension. When the descriptive system profile is linked to a ‘value set’, a single summary index value for health status is derived with scores that range from 0 to 1 (1 representing perfect health

and 0 representing the poorest health). A value set provides values (weights) for each health state description according to the preferences of the general population of a country/region. The VAS records the patient's personal perspective of their current health status on a vertical rating scale with scores ranging from 0 to 100, higher scores representing better quality-of-life.

*Coronary Physiology Measurements*

For the purposes of the primary and relevant secondary endpoints, coronary physiology data underwent post hoc adjudication by an independent core laboratory (CoreAalst BV, Aalst, Belgium). Each individual tracing was assessed for quality based on pre-specified criteria and received a binary decision regarding adequate quality for inclusion in the final analysis. The ratio of resting distal coronary to aortic pressure (Pd/Pa), fractional flow reserve (FFR), pullback pressure gradient (PPG), coronary flow reserve (CFR) and the index of microcirculatory resistance (IMR) were calculated independently from the corresponding recordings. Percentage change was defined as ([Post-PCI Value – Pre-PCI Value] / Pre-PCI Value x 100).

*Definition of Angina*

The process of defining residual angina presents its own challenges and alternative definitions could have been applied in the present study with a resultant variation in the reported incidence. For example, when considering angina at baseline, 71.9% of patients (187/260) had angina as defined by a patient-reported SAQ-Angina Frequency score < 100; 82.7% (215/260) had angina as defined by physician-adjudicated CCS Class 1 and above; and 90.8% (236/260) had angina as defined by a patient-reported SAQ-Summary Score < 100. The rates of residual angina as defined by SAQ-AF score < 100 and SAQ-SS score < 100 were 38.3% (88/230) and 62.2% (143/230) respectively. CCS Class at follow-up was not available.

**Figure S1. Change in FFR from pre- to post-PCI stratified by presence of angina 3 months post-PCI.**

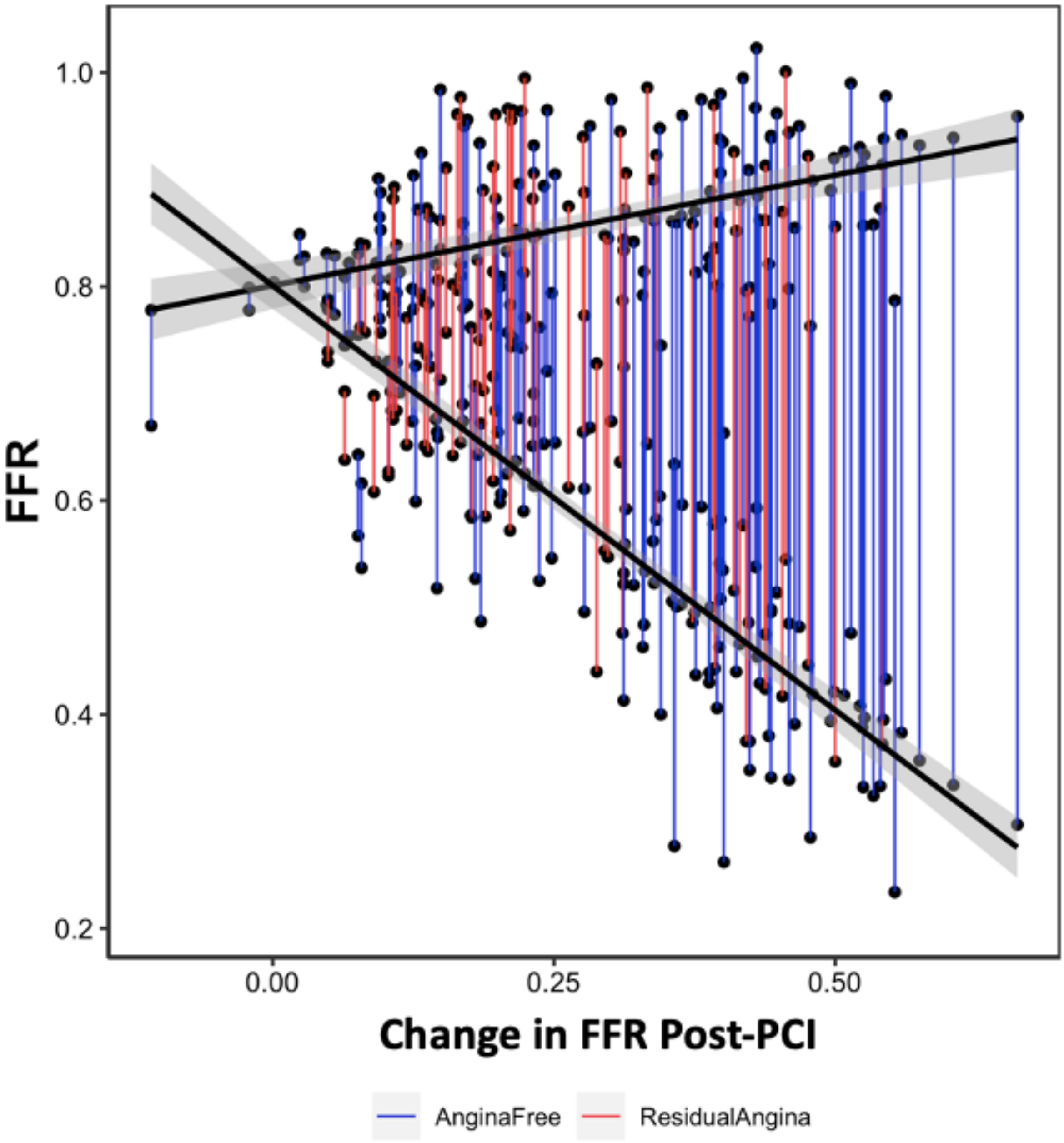


FFR=Fractional Flow Reserve; PCI=Percutaneous Coronary Intervention

**Table S1. Baseline clinical characteristics stratified by presence of baseline and post-PCI angina**

| Variables | Total | Baseline Angina + No Post-PCI Angina | Baseline Angina + Post-PCI Angina | No Baseline Angina + No Post-PCI Angina | No Baseline Angina + Post-PCI Angina | p-value |
|---|---|---|---|---|---|---|
| Number of patients, n (%) | 230 | 111 (48.3) | 83 (36.1) | 31 (13.5) | 5 (2.2) | |
| Male, n (%) | 202 (87.8) | 97 (87.4) | 70 (84.3) | 30 (96.8) | 5 (100) | 0.26 |
| Age years, mean (SD) | 60.9 ± 8.6 | 61.4 ± 8.2 | 61.4 ± 9.4 | 59 ± 7.2 | 54.8 ± 8.8 | 0.20 |
| BMI, mean (SD) | 29.8 ± 5.5 | 29.4 ± 5.1 | 30.8 ± 6.4 | 27.5 ± 3.4 | 33.4 ± 5.6 | **0.01** |
| Family history of CAD, n (%) | 153 (66.5) | 44 (66.7) | 55 (66.3) | 20 (64.5) | 4 (80) | 0.93 |
| Smoking status, n (%) | | | | | | 0.06 |
| Non-smoker | 72 (31.3) | 33 (29.7) | 23 (27.7) | 15 (48.4) | 1 (20) | |
| Current Smoker | 37 (16.1) | 15 (13.5) | 19 (22.9) | 1 (3.2) | 2 (40) | |
| Ex-smoker | 121 (52.6) | 63 (56.8) | 41 (49.4) | 15 (48.4) | 2 (40) | |
| Hypertension, n (%) | 103 (44.8) | 49 (44.1) | 44 (53) | 8 (25.8) | 2 (40) | 0.08 |
| Dyslipidemia, n (%) | 128 (55.7) | 70 (63.1) | 43 (51.8) | 14 (45.2) | 1 (20) | 0.07 |
| Heart Failure, n (%) | 53 (23) | 23 (20.7) | 19 (22.9) | 8 (25.8) | 3 (60) | 0.23 |
| Diabetes, n (%) | 39 (17) | 16 (14.4) | 19 (22.9) | 3 (9.7) | 1 (20) | 0.29 |
| Atrial Fibrillation, n (%) | 18 (7.8) | 4 (3.6) | 12 (14.5) | 2 (6.5) | 0 (0) | **0.04** |
| Chronic Kidney Disease, n (%) | 4 (1.7) | 1 (0.9) | 2 (2.4) | 1 (3.2) | 0 (0) | 0.76 |
| Indication for PCI, n (%) | | | | | | **0.002** |
| Stable Angina | 67 (29.1) | 36 (32.4) | 27 (32.5) | 4 (12.9) | 0 (0) | |
| ACS – UA/NSTEMI | 91 (39.6) | 52 (46.8) | 28 (33.7) | 9 (29) | 2 (40) | |
| Staged Completion of Revascularization | 72 (31.3) | 23 (20.7) | 28 (33.7) | 18 (58.1) | 3 (60) | |
| Previous MI | 83 (36.1) | 25 (22.5) | 37 (44.6) | 18 (58.1) | 3 (60) | **<0.001** |
| Previous PCI, n (%) | 86 (37.4) | 27 (24.3) | 38 (45.8) | 18 (58.1) | 3 (60) | **0.001** |
| Baseline CCS Class, n (%) | | | | | | **<0.001** |
| CCS 0 | 36 (15.7) | 0 (0) | 0 (0) | 31 (100) | 5 (100) | |
| CCS 1 | 56 (24.3) | 45 (40.5) | 11 (13.3) | 0 (0) | 0 (0) | |
| CCS 2 | 85 (37) | 44 (39.6) | 41 (49.4) | 0 (0) | 0 (0) | |
| CCS 3 | 52 (22.6) | 21 (18.9) | 31 (37.3) | 0 (0) | 0 (0) | |
| CCS 4 | 1 (0.4) | 1 (0.9) | 0 (0) | 0 (0) | 0 (0) | |
| Baseline SAQ-AF < 100 | 168 (73) | 84 (75.7) | 78 (94) | 5 (16.1) | 1 (20) | **<0.001** |
| Medications | | | | | | |
| Any antiplatelet, n (%) | 223 (97) | 111 (100) | 77 (92.8) | 30 (96.8) | 5 (100) | **0.04** |
| DAPT, n (%) | 160 (69.6) | 71 (64) | 58 (69.9) | 26 (83.9) | 5 (100) | 0.08 |
| Oral anticoagulant, n (%) | 16 (7) | 3 (2.7) | 13 (15.7) | 0 (0) | 0 (0) | **0.001** |
| Statins, n (%) | 221 (96.1) | 108 (97.3) | 77 (92.8) | 31 (100) | 5 (100) | 0.29 |

| | | | | | | |
|---|---|---|---|---|---|---|
| ACEI, n (%) | 150 (65.2) | 69 (62.2) | 50 (60.2) | 26 (83.9) | 5 (100) | **0.03** |
| ARB, n (%) | 23 (10) | 8 (7.2) | 13 (15.7) | 2 (6.5) | 0 (0) | 0.18 |
| Diuretics, n (%) | 27 (11.7) | 9 (8.1) | 15 (18.1) | 3 (9.7) | 0 (0) | 0.14 |
| Number of Anti-anginal Agents, mean (SD) | 1.73 ± 0.82 | 1.75 ± 0.76 | 2.02 ± 0.83 | 0.97 ± 0.55 | 1.00 ± 0.00 | **<0.001** |
| Beta-blocker, n (%) | 208 (90.4) | 100 (90.1) | 77 (92.8) | 26 (83.9) | 5 (100) | 0.46 |
| Calcium channel blocker, n (%) | 45 (19.6) | 25 (22.5) | 19 (22.9) | 1 (3.2) | 0 (0) | 0.05 |
| Nicorandil, n (%) | 17 (7.4) | 8 (7.2) | 9 (10.8) | 0 (0) | 0 (0) | 0.23 |
| Ivabradine, n (%) | 4 (1.7) | 2 (1.8) | 2 (2.4) | 0 (0) | 0 (0) | 0.84 |
| Oral Nitrates, n (%) | 66 (28.7) | 31 (27.9) | 33 (39.8) | 2 (6.5) | 0 (0) | **0.002** |
| Frequency of GTN spray use, n (%) | | | | | | **<0.001** |
| None | 115 (50) | 59 (53.2) | 21 (25.3) | 30 (96.8) | 5 (100) | |
| Daily | 29 (12.6) | 11 (9.9) | 18 (21.7) | 0 (0) | 0 (0) | |
| Weekly | 61 (26.5) | 27 (24.3) | 34 (41) | 0 (0) | 0 (0) | |
| Monthly | 25 (10.9) | 14 (12.6) | 10 (12) | 1 (3.2) | 0 (0) | |

ACEI=Angiotensin-Converting Enzyme Inhibitor; ACS-UA/NSTEMI=Acute Coronary Syndrome-Unstable Angina/Non-ST segment Elevation Myocardial Infarction; ARB=Angiotensin-Receptor Blocker; BMI=Body Mass Index; CCS=Canadian Cardiovascular Society; DAPT=Dual Anti-Platelet Therapy; GTN=Glyceryl Trinitrate; PCI=Percutaneous Coronary Intervention

**Table S2. Procedural and coronary physiology characteristics stratified by presence of baseline and post-PCI angina**

| Variables | Overall | Baseline Angina + No Post-PCI Angina | Baseline Angina + Post-PCI Angina | No Baseline Angina + No Post-PCI Angina | No Baseline Angina + Post-PCI Angina | p-value |
|---|---|---|---|---|---|---|
| Number of patients, n (%) | 230 | 111 (48.3) | 83 (36.1) | 31 (13.5) | 5 (2.2) | |
| Target Vessel, n (%) | | | | | | 0.67 |
| LAD | 130 (56.5) | 63 (56.8) | 45 (54.2) | 20 (64.5) | 2 (40) | |
| Non-LAD | 100 (43.5) | 48 (43.2) | 38 (45.8) | 11 (35.5) | 3 (60) | |
| Diameter stenosis (%), mean ± SD | 65.9 ± 15.4 | 67.8 ± 15.3 | 63.8 ± 15.2 | 64.1 ± 15.0 | 69.4 ± 19.0 | 0.27 |
| Lesion length, mean ± SD | 12.2 ± 6.0 | 12.6 ± 6.5 | 12.1 ± 5.4 | 11.5 ± 5.9 | 11.6 ± 6.4 | 0.82 |
| AHA/ACC Lesion type, n (%) | | | | | | 0.11 |
| A | 40 (17.4) | 22 (19.8) | 9 (10.8) | 9 (29) | 0 (0) | |
| B1 | 83 (36.1) | 39 (35.1) | 38 (45.8) | 5 (16.1) | 1 (20) | |
| B2 | 91 (39.6) | 42 (37.8) | 31 (37.3) | 15 (48.4) | 3 (60) | |
| C | 16 (7) | 8 (7.2) | 5 (6) | 2 (6.5) | 1 (20) | |
| SYNTAX score, mean ± SD | 11.2 ± 8.1 | 11.5 ± 8.3 | 10.5 ± 7.6 | 11.9 ± 8.8 | 8.8 ± 9.1 | 0.70 |
| BCIS Jeopardy score, mean ± SD | 5.0 ± 3.0 | 5.0 ± 3.2 | 5.1 ± 2.9 | 4.4 ± 2.7 | 4.0 ± 2.4 | 0.57 |
| Pre-PCI Pd/Pa, mean ± SD | 0.75 ± 0.18 | 0.71 ± 0.19 | 0.79 ± 0.15 | 0.80 ± 0.18 | 0.74 ± 0.12 | **0.004** |
| Pre-PCI FFR, mean ± SD | 0.58 ± 0.14 | 0.53 ± 0.15 | 0.62 ± 0.12 | 0.65 ± 0.14 | 0.53 ± 0.12 | **<0.001** |
| Pre-PCI resting TT | 1.13 ± 0.44 | 1.15 ± 0.41 | 1.07 ± 0.47 | 1.17 ± 0.48 | 1.21 ± 0.51 | 0.58 |
| Pre-PCI hyperemic TT | 0.69 ± 0.40 | 0.80 ± 0.44 | 0.58 ± 0.34 | 0.59 ± 0.27 | 0.85 ± 0.43 | **0.001** |
| Pre-PCI CFR, mean ± SD | 1.9 ± 0.9 | 1.7 ± 0.8 | 2.2 ± 1.0 | 2.1 ± 0.7 | 1.6 ± 0.6 | **0.004** |
| Pre-PCI IMR, mean ± SD | 28 ± 12 | 30 ± 12 | 26 ± 13 | 26 ± 10 | 32 ± 13 | 0.29 |
| Pre-PCI IMRc, mean ± SD | 21 ± 11 | 20 ± 10 | 21 ± 12 | 22 ± 10 | 20 ± 7 | 0.88 |
| Pre-dilatation, n (%) | 230 (100) | 111 (100.0) | 83 (100.0) | 31 (100.0) | 5 (100) | NA |
| Post-dilatation, n (%) | 225 (97.8) | 108 (97.3) | 82 (98.8) | 30 (96.8) | 5 (100) | 0.85 |
| Intravascular imaging, n (%) | 36 (15.7) | 19 (17.1) | 12 (14.5) | 3 (9.7) | 2 (40) | 0.34 |
| PIOS Applied, n (%) | 33 (14.3) | 19 (17.1) | 9 (10.8) | 5 (16.1) | 0 (0) | 0.49 |
| Number of stents (per vessel), mean ± SD | 1.46 ± 0.66 | 1.43 ± 0.64 | 1.46 ± 0.67 | 1.52 ± 0.77 | 1.60 ± 0.55 | 0.89 |
| Stent diameter (mm), mean ± SD | 3.22 ± 0.43 | 3.22 ± 0.41 | 3.23 ± 0.43 | 3.06 ± 0.43 | 3.70 ± 0.45 | **0.01** |
| Total stent length (mm), mean ± SD | 41.8 ± 20.1 | 41.6 ± 20.1 | 43.0 ± 21.2 | 39.5 ± 18.3 | 38.4 ± 11.8 | 0.84 |
| Residual diameter stenosis, mean ± SD | 14.2 ± 8.6 | 14.0 ± 8.8 | 14.9 ± 8.6 | 13.4 ± 9.0 | 9.8 ± 4.1 | 0.54 |

| | | | | | | |
|---|---|---|---|---|---|---|
| Residual SYNTAX score, mean ± SD | 2.31 ± 4.38 | 2.31 ± 4.18 | 2.52 ± 4.83 | 1.97 ± 3.77 | 0 ± 0 | 0.61 |
| Post-PCI Pd/Pa, mean ± SD | 0.94 ± 0.05 | 0.94 ± 0.06 | 0.94 ± 0.05 | 0.93 ± 0.05 | 0.94 ± 0.05 | 0.89 |
| Post-PCI FFR, mean ± SD | 0.86 ± 0.08 | 0.86 ± 0.08 | 0.85 ± 0.08 | 0.84 ± 0.10 | 0.88 ± 0.07 | 0.45 |
| Post-PCI resting TT | 0.91 ± 0.42 | 0.92 ± 0.43 | 0.84 ± 0.37 | 1.06 ± 0.47 | 1.10 ± 0.43 | 0.06 |
| Post-PCI hyperemic TT | 0.32 ± 0.21 | 0.32 ± 0.23 | 0.32 ± 0.21 | 0.33 ± 0.16 | 0.26 ± 0.12 | 0.92 |
| Post-PCI CFR, mean ± SD | 3.4 ± 1.9 | 3.4 ± 1.8 | 3.3 ± 2.2 | 3.7 ± 1.7 | 4.6 ± 1.7 | 0.45 |
| Post-PCI IMR | 22 ± 17 | 22 ± 19 | 22 ± 16 | 20 ± 10 | 17 ± 8 | 0.86 |
| Post-PCI IMRc | 21 ± 16 | 21 ± 19 | 22 ± 16 | 19 ± 10 | 17 ± 8 | 0.85 |
| Delta Pd/Pa | 0.19 ± 0.18 | 0.24 ± 0.18 | 0.14 ± 0.15 | 0.13 ± 0.19 | 0.20 ± 0.08 | **0.001** |
| % Delta Pd/Pa, mean ± SD | 37.0 ± 57.4 | 47.3 ± 55 | 25.2 ± 45.6 | 32.6 ± 87.4 | 29.3 ± 13.9 | 0.09 |
| Delta FFR, mean ± SD | 0.28 ± 0.15 | 0.33 ± 0.16 | 0.22 ± 0.12 | 0.18 ± 0.14 | 0.36 ± 0.10 | **<0.001** |
| % Delta FFR, mean ± SD | 58.3 ± 46.5 | 74.9 ± 50.4 | 40.8 ± 32.6 | 35.1 ± 38.3 | 73.2 ± 34.7 | **<0.001** |
| Delta resting TT | -0.22 ± 0.46 | -0.23 ± 0.50 | -0.22 ± 0.42 | -0.13 ± 0.36 | -0.27 ± 0.58 | 0.77 |
| % Delta resting TT, mean ± SD | -11.8 ± 43.2 | -12.1 ± 47.5 | -12.9 ± 41.0 | -8.0 ± 33.5 | -10.9 ± 50.9 | 0.96 |
| Delta hyperemic TT | -0.38 ± 0.41 | -0.49 ± 0.44 | -0.27 ± 0.37 | -0.27 ± 0.28 | -0.64 ± 0.39 | **0.001** |
| % Delta hyperemic TT, mean ± SD | -43.3 ± 41.9 | -52.2 ± 31 | -33.0 ± 53.3 | -36.5 ± 33.6 | -69.6 ± 18.5 | **0.01** |
| Delta CFR, mean ± SD | 1.5 ± 1.8 | 1.8 ± 1.9 | 1.2 ± 1.8 | 1.4 ± 1.4 | 3.2 ± 1.5 | 0.06 |
| % Delta CFR, mean ± SD | 103.8 ± 131 | 136.4±158.7 | 68.0 ± 90.8 | 75.4 ± 83.7 | 208.2±100.9 | **0.001** |
| Delta IMR | -7 ± 16 | -9 ± 15 | -4 ± 18 | -7 ± 11 | -18 ± 9 | 0.14 |
| % Delta IMR, mean ± SD | -16.4 ± 66.5 | -25.1 ± 47.2 | -3.3 ± 89.1 | -17 ± 50 | -53.6 ± 13.2 | 0.14 |
| Delta IMRc | -1 ± 15 | -1 ± 16 | 0 ± 17 | -3 ± 10 | -6 ± 4 | 0.75 |
| % Delta IMRc, mean ± SD | 13.5 ± 125.5 | 14.3 ± 140.9 | 18.8 ± 125.0 | 3.6 ± 75 | -30.1 ± 13.0 | 0.86 |
| Periprocedural MI, n (%) | 7 (3) | 1 (1) | 4 (5) | 2 (6) | 0 (0) | |

ACC=American College of Cardiology; AHA=American Heart Association; BCIS=British Cardiovascular Intervention Society; CFR=Coronary Flow Reserve; FFR=Fractional Flow Reserve; IMR=Index of Microcirculatory Resistance; IMRc=Index of Microcirculatory Resistance corrected for epicardial stenosis (Yong's formula); MI=Myocardial Infaction; Pd/Pa=Ratio of resting distal coronary to aortic pressure; PIOS=Physiology-guided Optimization Protocol; PPG=Pullback Pressure Gradient; SD=Standard Deviation; TT=Transit Time;

**Table S3. Baseline, Follow-Up and Change in SAQ-7 scores stratified by presence of baseline (CCS class ≥ 1) and post-PCI angina (SAQ-AF score < 100)**

| Variables | Overall | Baseline Angina + No Post-PCI Angina | Baseline Angina + Post-PCI Angina | No Baseline Angina + No Post-PCI Angina | No Baseline Angina + Post-PCI Angina | p-value |
|---|---|---|---|---|---|---|
| n (%) | 230 | 111 (48.3) | 83 (36.1) | 31 (13.5) | 5 (2.2) | |
| **Baseline SAQ-7** | | | | | | |
| Physical Limitation score, mean ± SD | 68.61 ± 26.66 | 69.72 ± 24.17 | 54.64 ± 23.86 | 99.69 ± 1.60 | 100 ± 0 | **<0.001** |
| Physical Limitation categories | | | | | | **<0.001** |
| Very Poor to Poor, n (%) | 7/208 (3.4) | 2/97 (2.1) | 5/79 (6.3) | 0/27 (0) | 0/5 (0) | |
| Poor to Fair, n (%) | 38/208 (18.3) | 14/97 (14.4) | 24/79 (30.4) | 0/27 (0) | 0/5 (0) | |
| Fair to Good, n (%) | 58/208 (27.9) | 31/97 (32) | 27/79 (34.2) | 0/27 (0) | 0/5 (0) | |
| Good to Excellent, n (%) | 105/208 (50.5) | 50/97 (51.5) | 23/79 (29.1) | 27/27 (100) | 5/5 (100) | |
| Angina Frequency score, mean ± SD | 68.83 ± 28.73 | 70.27 ± 27.81 | 54.46 ± 26.05 | 97.74 ± 5.60 | 96.00 ± 8.94 | **<0.001** |
| Angina Frequency categories | | | | | | **<0.001** |
| Daily, n (%) | 35 (15.2) | 13 (11.7) | 22 (26.5) | 0 (0) | 0 (0) | |
| Weekly, n (%) | 63 (27.4) | 30 (27) | 33 (39.8) | 0 (0) | 0 (0) | |
| Monthly, n (%) | 70 (30.4) | 41 (36.9) | 23 (27.7) | 5 (16.1) | 1 (20) | |
| None, n (%) | 62 (27) | 27 (24.3) | 5 (6) | 26 (83.9) | 4 (80) | |
| Quality of Life score, mean ± SD | 48.75 ± 30.54 | 47.64 ± 27.88 | 34.79 ± 25.08 | 86.29 ± 18.64 | 72.50 ± 28.50 | **<0.001** |
| Quality of Life categories | | | | | | **<0.001** |
| Very Poor to Poor, n (%) | 46 (20) | 18 (16.2) | 28 (33.7) | 0 (0) | 0 (0) | |
| Poor to Fair, n (%) | 69 (30) | 38 (34.2) | 30 (36.1) | 0 (0) | 1 (20) | |
| Fair to Good, n (%) | 50 (21.7) | 27 (24.3) | 16 (19.3) | 6 (19.4) | 1 (20) | |
| Good to Excellent, n (%) | 65 (28.3) | 28 (25.2) | 9 (10.8) | 25 (80.6) | 3 (60) | |
| Summary Score, mean ± SD | 62.10 ± 25.30 | 62.57 ± 22.55 | 47.83 ± 20.86 | 94.20 ± 7.86 | 89.50 ± 11.75 | **<0.001** |
| Summary Score categories | | | | | | **<0.001** |
| Very Poor to Poor, n (%) | 17 (7.4) | 6 (5.4) | 11 (13.3) | 0 (0) | 0 (0) | |
| Poor to Fair, n (%) | 61 (26.5) | 25 (22.5) | 36 (43.4) | 0 (0) | 0 (0) | |
| Fair to Good, n (%) | 69 (30) | 41 (36.9) | 27 (32.5) | 0 (0) | 1 (20) | |

| | | | | | | |
|---|---|---|---|---|---|---|
| Good to Excellent, n (%) | 83 (36.1) | 39 (35.1) | 9 (10.8) | 31 (100) | 4 (80) | |
| | | | | | | |
| **Follow-up SAQ** | | | | | | |
| Physical Limitation score, mean ± SD | 81.69 ± 25.73 | 90.84 ± 19.13 | 66.17 ± 28.24 | 92.71 ± 18.11 | 79.17 ± 22.05 | **<0.001** |
| Physical Limitation categories | | | | | | **<0.001** |
| Very Poor to Poor, n (%) | 10/205 (4.9) | 2/101 (2) | 7/76 (9.2) | 1/24 (4.2) | 0/4 (0) | |
| Poor to Fair, n (%) | 15/205 (7.3) | 3/101 (3) | 12/76 (15.8) | 0/24 (0) | 0/4 (0) | |
| Fair to Good, n (%) | 24/205 (11.7) | 7/101 (6.9) | 15/76 (19.7) | 1/24 (4.2) | 1/4 (25) | |
| Good to Excellent, n (%) | 156/205 (76.1) | 89/101 (88.8) | 42/76 (55.3) | 22/24 (91.7) | 3/4 (75) | |
| Angina Frequency score, mean ± SD | 87.65 ± 19.84 | 100 ± 0 | 66.99 ± 19.92 | 100 ± 0 | 80.00 ± 7.07 | **<0.001** |
| Angina Frequency categories | | | | | | **<0.001** |
| Daily, n (%) | 5 (2.2) | 0 (0) | 5 (6) | 0 (0) | 0 (0) | |
| Weekly, n (%) | 32 (13.9) | 0 (0) | 32 (38.6) | 0 (0) | 0 (0) | |
| Monthly, n (%) | 51 (22.2) | 0 (0) | 46 (55.4) | 0 (0) | 5 (100) | |
| None, n (%) | 142 (61.7) | 111 (100) | 0 (0) | 31 (100) | 0 (0) | |
| Quality of Life score, mean ± SD | 80.11 ± 27.12 | 93.02 ± 14.66 | 56.48 ± 28.78 | 95.97 ± 10.89 | 87.50 ± 8.84 | **<0.001** |
| Quality of Life categories | | | | | | **<0.001** |
| Very Poor to Poor, n (%) | 12 (5.2) | 0 (0) | 12 (14.5) | 0 (0) | 0 (0) | |
| Poor to Fair, n (%) | 19 (8.3) | 2 (1.8) | 17 (20.5) | 0 (0) | 0 (0) | |
| Fair to Good, n (%) | 28 (12.2) | 7 (6.3) | 20 (24.1) | 1 (3.2) | 0 (0) | |
| Good to Excellent, n (%) | 171 (74.3) | 102 (91.9) | 34 (41) | 30 (96.8) | 5 (100) | |
| Summary Score, mean ± SD | 83.24 ± 21.47 | 94.71 ± 9.02 | 62.87 ± 22.04 | 96.77 ± 7.45 | 82.94 ± 9.95 | **<0.001** |
| Summary Score categories | | | | | | **<0.001** |
| Very Poor to Poor, n (%) | 3 (1.3) | 0 (0) | 3 (3.6) | 0 (0) | 0 (0) | |
| Poor to Fair, n (%) | 22 (9.6) | 0 (0) | 22 (26.5) | 0 (0) | 0 (0) | |
| Fair to Good, n (%) | 36 (15.7) | 6 (5.4) | 28 (33.7) | 1 (3.2) | 1 (20) | |
| Good to Excellent, n (%) | 169 (73.5) | 105 (94.6) | 30 (36.1) | 30 (96.8) | 4 (80) | |
| **Change in SAQ Scores** | | | | | | |
| Change in Physical Limitation score, mean ± SD | 13.81 ± 27.64 | 22.55 ± 27.60 | 9.87 ± 26.86 | -4.17 ± 9.17 | -20.83 ± 22.05 | **<0.001** |

| | | | | | | |
|---|---|---|---|---|---|---|
| Change in Angina Frequency score, mean ± SD | 18.83 ± 28.13 | 29.73 ± 27.81 | 12.53 ± 27.84 | 2.26 ± 5.60 | -16.00 ± 11.40 | **<0.001** |
| Change in Quality of Life score, mean ± SD | 31.36 ± 30.75 | 45.38 ± 27.43 | 21.69 ± 30.99 | 9.68 ± 15.38 | 15.00 ± 24.04 | **<0.001** |
| Change in Summary Score, mean ± SD | 21.14 ± 24.73 | 32.14 ± 23.30 | 15.04 ± 23.81 | 2.58 ± 8.18 | -6.56 ± 14.23 | **<0.001** |

SAQ=Seattle Angina Questionnaire

**Table S4. Baseline and follow-up EQ-5D-5L scores stratified by stratified by presence of baseline (CCS class ≥ 1) and post-PCI angina (SAQ-AF score < 100)**

| Variables | Total | Baseline Angina + No Post-PCI Angina | Baseline Angina + Post-PCI Angina | No Baseline Angina + No Post-PCI Angina | No Baseline Angina + Post-PCI Angina | p-value |
|---|---|---|---|---|---|---|
| **Baseline EQ-5D-5L** | | | | | | |
| Mobility score, mean ± SD | 1.8 ± 1.0 | 1.7 ± 0.9 | 2.3 ± 1.0 | 1.2 ± 0.8 | 1.0 ± 0.0 | **<0.001** |
| Self-care score, mean ± SD | 1.2 ± 0.5 | 1.1 ± 0.4 | 1.4 ± 0.6 | 1.0 ± 0.2 | 1.0 ± 0.0 | **<0.001** |
| Usual activities score, mean ± SD | 2.2 ± 1.2 | 2.2 ± 1.2 | 2.6 ± 1.1 | 1.5 ± 0.9 | 1.0 ± 0.0 | **<0.001** |
| Pain score, mean ± SD | 2.0 ± 1.0 | 1.9 ± 0.8 | 2.5 ± 1.0 | 1.3 ± 0.8 | 1.2 ± 0.4 | **<0.001** |
| Anxiety and depression score, mean ± SD | 1.8 ± 0.9 | 1.6 ± 0.7 | 2.2 ± 0.9 | 1.3 ± 0.8 | 1.8 ± 0.8 | **<0.001** |
| Visual Analogue Scale, mean ± SD | 69.8 ± 18.2 | 72.2 ± 16.5 | 62.7 ± 18.7 | 81.2 ± 15.0 | 67.2 ± 19.2 | **<0.001** |
| EQ-5D-5L index, mean ± SD | 0.78 ± 0.20 | 0.81 ± 0.15 | 0.68 ± 0.22 | 0.91 ± 0.15 | 0.94 ± 0.06 | **<0.001** |
| **Follow-Up EQ-5D-5L** | | | | | | |
| Mobility score, mean ± SD | 1.6 ± 1.0 | 1.4 ± 0.8 | 2.1 ± 1.2 | 1.2 ± 0.6 | 1.6 ± 0.6 | **<0.001** |
| Self-care score, mean ± SD | 1.3 ± 0.7 | 1.1 ± 0.5 | 1.6 ± 0.9 | 1.1 ± 0.4 | 1.0 ± 0.0 | **<0.001** |
| Usual activities score, mean ± SD | 1.7 ± 1.0 | 1.4 ± 0.8 | 2.4 ± 1.1 | 1.3 ± 0.7 | 1.4 ± 0.6 | **<0.001** |
| Pain score, mean ± SD | 1.7 ± 0.9 | 1.4 ± 0.8 | 2.4 ± 0.9 | 1.3 ± 0.8 | 1.2 ± 0.4 | **<0.001** |
| Anxiety and depression score, mean ± SD | 1.7 ± 1.1 | 1.4 ± 0.8 | 2.3 ± 1.3 | 1.3 ± 0.5 | 1.8 ± 0.8 | **<0.001** |
| Visual Analogue Scale, mean ± SD | 76.9 ± 18.3 | 83.3 ± 12.4 | 65.3 ± 21.2 | 83.9 ± 12.4 | 79.6 ± 17.8 | **<0.001** |
| EQ-5D-5L index, mean ± SD | 0.82 ± 0.23 | 0.90 ± 0.17 | 0.68 ± 0.27 | 0.93 ± 0.14 | 0.88 ± 0.09 | **<0.001** |
| Change in Weighted Health Index | 0.04 ± 0.22 | 0.08 ± 0.20 | 0.00 ± 0.27 | 0.02 ± 0.09 | -0.05 ± 0.10 | **0.045** |

EQ-5D-5L: European Quality-of-Life–5 Dimensions–5 Levels questionnaire

**Table S5. Predictors of No Post-PCI Angina in Patients with Baseline Angina (n=194)**

| | **Univariate HR (95% CI)** | **p value** | **Multivariate HR (95% CI)** | **p value** |
|---|---|---|---|---|
| BMI | 0.96 (0.91 to 1.01) | 0.09 | 0.95 (0.89 to 1.02) | 0.13 |
| Current Smoker | 0.55 (0.23 to 1.3) | 0.17 | 0.72 (0.25 to 2.09) | 0.55 |
| Ex-smoker | 1.07 (0.55 to 2.07) | 0.84 | 1.31 (0.57 to 3.03) | 0.52 |
| Atrial Fibrillation | 0.22 (0.06 to 0.66) | **0.01** | 0.42 (0.1 to 1.6) | 0.21 |
| Previous PCI | 0.38 (0.2 to 0.7) | **0.002** | 0.5 (0.22 to 1.12) | 0.09 |
| Delta FFR (0.10 increments) | 1.68 (1.34 to 2.16) | **<0.001** | 1.53 (1.17 to 2.02) | **0.002** |
| Periprocedural MI | 0.18 (0.01 to 1.24) | 0.13 | 0.23 (0.01 to 1.82) | 0.22 |
| Cardiac Rehab Attendance | 0.49 (0.23 to 1.01) | 0.054 | 0.72 (0.3 to 1.74) | 0.46 |

BMI=Body Mass Index; FFR=Fractional Flow Reserve; HR=Hazard Ratio; MI=Myocardial Infarction; PCI=Percutaneous Coronary Intervention

**Table S6. Baseline, Follow-Up and Change in SAQ-7 scores stratified by presence of post-PCI angina (SAQ-AF score < 100 at follow-up)**

| Variables | Overall | No Post-PCI Angina | Post-PCI Angina | p-value |
|---|---|---|---|---|
| n | 230 | 142 | 88 | |
| **Baseline SAQ-7** | | | | |
| Physical Limitation score, mean ± SD | 68.61±26.66 | 76.24±24.71 | 57.34±25.53 | **<0.001** |
| Physical Limitation categories | | | | **<0.001** |
| Very Poor to Poor, n (%) | 7/208 (3.4) | 2/124 (1.6) | 5/84 (6) | |
| Poor to Fair, n (%) | 38/208 (18.3) | 14/124 (11.3) | 24/84 (28.6) | |
| Fair to Good, n (%) | 58/208 (27.9) | 31/124 (25) | 27/84 (32.1) | |
| Good to Excellent, n (%) | 105/208 (50.5) | 77/124 (62.1) | 28/84 (33.3) | |
| Angina Frequency score, mean ± SD | 68.83±28.73 | 76.27±27.20 | 56.82± 7.15 | **<0.001** |
| Angina Frequency categories | | | | **<0.001** |
| Daily, n (%) | 35 (15.2) | 13 (9.2) | 22 (25) | |
| Weekly, n (%) | 63 (27.4) | 30 (21.1) | 33 (37.5) | |
| Monthly, n (%) | 70 (30.4) | 46 (32.4) | 24 (27.3) | |
| None, n (%) | 62 (27) | 53 (37.3) | 9 (10.2) | |
| Quality of Life score, mean ± SD | 48.75±30.54 | 56.07±30.61 | 36.93±26.59 | **<0.001** |
| Quality of Life categories | | | | **<0.001** |
| Very Poor to Poor, n (%) | 46 (20) | 18 (12.7) | 28 (31.8) | |
| Poor to Fair, n (%) | 69 (30) | 38 (26.8) | 31 (35.2) | |
| Fair to Good, n (%) | 50 (21.7) | 33 (23.2) | 17 (19.3) | |
| Good to Excellent, n (%) | 65 (28.3) | 53 (37.3) | 12 (13.6) | |
| Summary Score, mean ± SD | 62.10±25.30 | 69.48±24.12 | 50.20±22.59 | **<0.001** |
| Summary Score categories | | | | **<0.001** |
| Very Poor to Poor, n (%) | 17 (7.4) | 6 (4.2) | 11 (12.5) | |
| Poor to Fair, n (%) | 61 (26.5) | 25 (17.6) | 36 (40.9) | |
| Fair to Good, n (%) | 69 (30) | 41 (28.9) | 28 (31.8) | |
| Good to Excellent, n (%) | 83 (36.1) | 70 (49.3) | 13 (14.8) | |

| | | | | |
|---|---|---|---|---|
| **Follow-up SAQ** | | | | |
| Physical Limitation score, mean ± SD | 81.69±25.73 | 91.20±18.88 | 66.82±27.99 | **<0.001** |
| Physical Limitation categories | | | | **<0.001** |
| Very Poor to Poor, n (%) | 10/205 (4.9) | 3/125 (2.4) | 7/80 (8.8) | |
| Poor to Fair, n (%) | 15/205 (7.3) | 3/125 (2.4) | 12/80 (15) | |
| Fair to Good, n (%) | 24/205 (11.7) | 8/125 (6.4) | 16/80 (20) | |
| Good to Excellent, n (%) | 156/205 (76.1) | 111/125 (88.8) | 45/80 (56.3) | |
| Angina Frequency score, mean ± SD | 87.65±19.84 | 100±0 | 67.73±19.64 | **<0.001** |
| Angina Frequency categories | | | | **<0.001** |
| Daily, n (%) | 5 (2.2) | 0 | 5 (5.7) | |
| Weekly, n (%) | 32 (13.9) | 0 | 32 (36.4) | |
| Monthly, n (%) | 51 (22.2) | 0 | 51 (58) | |
| None, n (%) | 142 (61.7) | 142 (100) | 0 | |
| Quality-of-Life score, mean ± SD | 80.11±27.16 | 93.66±13.95 | 58.24±28.92 | <0.001 |
| Quality-of-Life categories | | | | **<0.001** |
| Very Poor to Poor, n (%) | 12 (5.2) | 0 | 12 (13.6) | |
| Poor to Fair, n (%) | 19 (8.3) | 2 (1.4) | 17 (19.3) | |
| Fair to Good, n (%) | 28 (12.2) | 8 (5.6) | 20 (22.7) | |
| Good to Excellent, n (%) | 171 (74.3) | 132 (93) | 39 (44.3) | |
| Summary Score, mean ± SD | 83.24±21.47 | 95.16±8.72 | 64.01±22 | **<0.001** |
| Summary Score categories | | | | **<0.001** |
| Very Poor to Poor, n (%) | 3 (1.3) | 0 | 3 (3.4) | |
| Poor to Fair, n (%) | 22 (9.6) | 0 | 22 (25) | |
| Fair to Good, n (%) | 36 (15.7) | 7 (4.9) | 29 (33) | |
| Good to Excellent, n (%) | 169 (73.5) | 135 (95.1) | 34 (38.6) | |
| **Change in SAQ Scores** | | | | |
| Change in Physical Limitation score, mean ± SD | 13.81±27.64 | 17.69±27.27 | 8.28±27.38 | **0.02** |
| Change in Angina Frequency score, mean ± SD | 18.83±28.13 | 23.73±27.20 | 10.91 ± 27.94 | **<0.001** |
| Change in Quality-of-Life score, mean ± SD | 31.36±30.75 | 37.59±29.27 | 21.31±30.57 | **<0.001** |

| | | | | |
|---|---|---|---|---|
| Change in Summary Score, mean ± SD | 21.14±24.73 | 25.68±24.25 | 13.81±23.85 | **<0.001** |

SAQ=Seattle Angina Questionnaire

**Table S7. Baseline and follow-up EQ-5D-5L scores stratified by presence of post-PCI angina (SAQ-AF score < 100 at follow-up)**

| Variables | Total | No Post-PCI Angina | Post-PCI Angina | p-value |
|---|---|---|---|---|
| **Baseline EQ-5D-5L** | | | | |
| Mobility score, mean ± SD | 1.8 ± 1.0 | 1.6 ± 0.9 | 2.2 ± 1.0 | **<0.001** |
| Self-care score, mean ± SD | 1.2 ± 0.5 | 1.1 ± 0.3 | 1.4 ± 0.6 | **<0.001** |
| Usual activities score, mean ± SD | 2.2 ± 1.2 | 2.0 ± 1.1 | 2.6 ± 1.1 | **<0.001** |
| Pain score, mean ± SD | 2.0 ± 1.0 | 1.7 ± 0.9 | 2.4 ± 1.0 | **<0.001** |
| Anxiety and depression score, mean ± SD | 1.8 ± 0.9 | 1.6 ± 0.8 | 2.2 ± 0.9 | **<0.001** |
| Visual Analogue Scale, mean ± SD | 69.8 ± 18.2 | 74.2 ± 16.6 | 62.9 ± 18.7 | **<0.001** |
| EQ-5D-5L index, mean ± SD | 0.78 ± 0.20 | 0.84 ± 0.15 | 0.69 ± 0.22 | **<0.001** |
| **Follow-Up EQ-5D-5L** | | | | |
| Mobility score, mean ± SD | 1.6 ± 1.0 | 1.3 ± 0.8 | 2.1 ± 1.1 | **<0.001** |
| Self-care score, mean ± SD | 1.3 ± 0.7 | 1.1 ± 0.4 | 1.6 ± 0.9 | **<0.001** |
| Usual activities score, mean ± SD | 1.7 ± 1.0 | 1.4 ± 0.7 | 2.3 ± 1.1 | **<0.001** |
| Pain score, mean ± SD | 1.7 ± 0.9 | 1.4 ± 0.8 | 2.3 ± 0.9 | **<0.001** |
| Anxiety and depression score, mean ± SD | 1.7 ± 1.1 | 1.4 ± 0.7 | 2.3 ± 1.3 | **<0.001** |
| Visual Analogue Scale, mean ± SD | 76.9 ±18.3 | 83.5 ±12.4 | 66.2 ± 21.2 | **<0.001** |
| EQ-5D-5L index, mean ± SD | 0.82 ± 0.23 | 0.91 ± 0.17 | 0.69 ± 0.26 | **<0.001** |
| Change in Weighted Health Index | 0.04 ± 0.22 | 0.07 ± 0.18 | -.001 ± 0.26 | **0.03** |

EQ-5D-5L: European Quality-of-Life–5 Dimensions–5 Levels questionnaire

**Table S8. Spearman correlation between FFR and follow-up Patient Reported Outcome Measure scores among patients with angina at baseline (CCS Class I and above)**

| Patient Reported Outcome Measure | FFR | Correlation Coefficient | P value |
|---|---|---|---|
| SAQ Physical Limitation Score (SAQ7-PL) | Baseline | -0.21 | **0.008** |
| | Post-PCI | 0.07 | 0.39 |
| | Absolute Change | 0.26 | **0.002** |
| | Percentage Change | 0.26 | **0.001** |
| | | | |
| SAQ Angina Frequency Score (SAQ7-AF) | Baseline | -0.31 | **<0.0001** |
| | Post-PCI | 0.07 | 0.35 |
| | Absolute Change | 0.35 | **<0.0001** |
| | Percentage Change | 0.36 | **<0.0001** |
| | | | |
| SAQ Quality of Life Score (SAQ7-QL) | Baseline | -0.17 | **0.02** |
| | Post-PCI | 0.03 | 0.70 |
| | Absolute Change | 0.21 | **0.008** |
| | Percentage Change | 0.22 | **0.006** |
| | | | |
| SAQ Summary Score (SAQ7-SS) | Baseline | -0.26 | **0.0004** |
| | Post-PCI | 0.01 | 0.90 |
| | Absolute Change | 0.29 | **0.0002** |
| | Percentage Change | 0.30 | **<0.0001** |
| | | | |
| EQ-5D-5L UK Weighted Health State | Baseline | -0.15 | **0.04** |
| | Post-PCI | 0.02 | 0.80 |
| | Absolute Change | 0.20 | **0.011** |
| | Percentage Change | 0.21 | **0.008** |

CCS=Canadian Cardiovascular Society; EQ-5D-5L= European Quality-of-Life-5 Dimension-5 Level questionnaire; FFR=Fractional Flow Reserve; PCI=Percutaneous Coronary Intervention; SAQ=Seattle Angina Questionnaire.

**Table S9. Follow-Up PROM scores stratified by tertiles of FFR at baseline, post-PCI and percentage change among patients with angina at baseline (CCS class I and above)**

| **FFR** | | **Low** | | **Intermediate** | | **High** | | **p value** |
|---|---|---|---|---|---|---|---|---|
| | **Patient Reported Outcome** | **N** | **Score** | **N** | **Score** | **N** | **Score** | |
| Baseline | SAQ Physical Limitation Score | 55 | 83.79±25.68 | 53 | 81.05±24.98 | 51 | 75.82±24.80 | 0.30 |
| | SAQ Angina Frequency Score | 61 | 93.77±13.31 | 59 | 82.71±23.48 | 54 | 80.74±23.14 | **0.001** |
| | SAQ Quality of Life Score | 61 | 84.02±23.62 | 59 | 75.64±29.94 | 54 | 74.31±28.98 | 0.12 |
| | SAQ Summary Score | 61 | 87.60±17.75 | 59 | 79.17±24.47 | 54 | 77.02±23.61 | **0.02** |
| | EQ-5D-5L Health State (UK) | 61 | 0.84±0.25 | 59 | 0.78±0.27 | 54 | 0.79±0.24 | 0.35 |
| Post-PCI | SAQ Physical Limitation Score | 51 | 81.54±23.94 | 57 | 77.92±26.70 | 54 | 80.86±28.94 | 0.75 |
| | SAQ Angina Frequency Score | 58 | 84.48±21.70 | 62 | 86.29±20.74 | 59 | 87.29±19.99 | 0.76 |
| | SAQ Quality of Life Score | 58 | 77.59±29.22 | 62 | 76.41±26.31 | 59 | 77.54±29.89 | 0.97 |
| | SAQ Summary Score | 58 | 81.62±22.46 | 62 | 80.25±20.72 | 59 | 81.17±24.12 | 0.94 |
| | EQ-5D-5L Health State (UK) | 58 | 0.82±0.24 | 62 | 0.81±0.21 | 59 | 0.79±0.29 | 0.87 |
| Percentage Change | SAQ Physical Limitation Score | 47 | 71.81±30.07 | 48 | 83.16±24.76 | 52 | 85.90±23.89 | **0.02** |
| | SAQ Angina Frequency Score | 50 | 78.60±23.56 | 55 | 85.09±21.33 | 57 | 94.39±13.89 | **<0.001** |

| | | | | | | | | |
|---|---|---|---|---|---|---|---|---|
| | SAQ Quality of Life Score | 50 | 71.75±29.75 | 55 | 77.95±28.05 | 57 | 84.43±23.89 | 0.06 |
| | SAQ Summary Score | 50 | 74.51±24.20 | 55 | 81.45±22.38 | 57 | 88.23±18.29 | **0.006** |
| | EQ-5D-5L Health State (UK) | 50 | 0.76±0.26 | 55 | 0.81±0.24 | 57 | 0.84±0.26 | 0.29 |

FFR=Fractional Flow Reserve; PCI=Percutaneous Coronary Intervention; SAQ=Seattle Angina Questionnaire

**Table S10. Spearman correlation between CFR and follow-up Patient Reported Outcome Measure scores among patients with angina at baseline (CCS Class I and above) (n=194)**

| Patient Reported Outcome Measure | CFR | Correlation Coefficient | P value |
|---|---|---|---|
| SAQ Physical Limitation Score (SAQ7-PL) | Baseline | -0.08 | 0.32 |
| | Post-PCI | 0.10 | 0.21 |
| | Absolute Change | 0.18 | **0.02** |
| | Percentage Change | 0.21 | **0.008** |
| | | | |
| SAQ Angina Frequency Score (SAQ7-AF) | Baseline | -0.24 | 0.002 |
| | Post-PCI | 0.08 | 0.26 |
| | Absolute Change | 0.20 | **0.009** |
| | Percentage Change | 0.26 | **<0.001** |
| | | | |
| SAQ Quality of Life Score (SAQ7-QL) | Baseline | -0.13 | 0.10 |
| | Post-PCI | 0.03 | 0.64 |
| | Absolute Change | 0.12 | 0.12 |
| | Percentage Change | 0.16 | **0.036** |
| | | | |
| SAQ Summary Score (SAQ7-SS) | Baseline | -0.16 | 0.03 |
| | Post-PCI | 0.07 | 0.37 |
| | Absolute Change | 0.17 | **0.03** |
| | Percentage Change | 0.21 | **0.005** |
| | | | |
| EQ-5D-5L UK Weighted Health Index | Baseline | -0.06 | 0.40 |
| | Post-PCI | 0.09 | 0.21 |
| | Absolute Change | 0.17 | **0.025** |
| | Percentage Change | 0.20 | **0.01** |

CCS=Canadian Cardiovascular Society; CFR=Coronary Flow Reserve; EQ-5D-5L=European Quality-of-Life-5 Dimension-5 Level questionnaire; PCI=Percutaneous Coronary Intervention; SAQ=Seattle Angina Questionnaire.

**Table S11. PROM scores 3 months post-PCI stratified by tertiles of CFR at baseline, post-PCI and percentage change among patients with angina at baseline**

| **CFR** | | **Low** | | **Intermediate** | | **High** | | **p value** |
|---|---|---|---|---|---|---|---|---|
| | **Patient Reported Outcome** | **N** | **Score** | **N** | **Score** | **N** | **Score** | |
| Baseline | SAQ Physical Limitation Score | 52 | 81.57±26.58 | 53 | 78.77±27.23 | 54 | 78.32±26.59 | 0.80 |
| | SAQ Angina Frequency Score | 61 | 91.31±16.48 | 56 | 83.04±22.96 | 57 | 80.53±23.18 | **0.015** |
| | SAQ Quality of Life Score | 61 | 81.56±25.88 | 56 | 75.67±31.21 | 57 | 74.12±28.14 | 0.32 |
| | SAQ Summary Score | 61 | 85.00±19.86 | 56 | 78.70±25.58 | 57 | 77.65±21.99 | 0.16 |
| | EQ-5D-5L Health State (UK) | 61 | 0.80±0.27 | 56 | 0.84±0.19 | 57 | 0.77±0.27 | 0.34 |
| Post-PCI | SAQ Physical Limitation Score | 56 | 75.52±26.83 | 51 | 83.99±23.62 | 63 | 81.22±27.80 | 0.23 |
| | SAQ Angina Frequency Score | 62 | 83.55±21.36 | 61 | 85.41±23.49 | 64 | 88.75±16.57 | 0.36 |
| | SAQ Quality of Life Score | 62 | 75.81±30.52 | 61 | 77.05±30.21 | 64 | 79.88±24.05 | 0.71 |
| | SAQ Summary Score | 62 | 77.93±23.95 | 61 | 82.09±23.16 | 64 | 83.38±20.10 | 0.37 |
| | EQ-5D-5L Health State (UK) | 62 | 0.80±0.23 | 61 | 0.80±0.23 | 64 | 0.82±0.27 | 0.86 |
| Percentage Change | SAQ Physical Limitation Score | 50 | 70.58±27.00 | 51 | 86.93±22.25 | 52 | 80.61±27.89 | **0.007** |
| | SAQ Angina Frequency Score | 55 | 76.18±25.35 | 55 | 90.00±15.87 | 58 | 89.14±18.28 | **<0.001** |

| | | | | | | | | |
|---|---|---|---|---|---|---|---|---|
| | SAQ Quality of Life Score | 55 | 68.18±33.14 | 55 | 85.45±19.80 | 58 | 78.88±28.02 | **0.005** |
| | SAQ Summary Score | 55 | 71.90±25.71 | 55 | 87.38±16.11 | 58 | 82.58±22.47 | **0.006** |
| | EQ-5D-5L Health State (UK) | 55 | 0.72±0.25 | 55 | 0.88±0.17 | 58 | 0.81±0.27 | **<0.001** |

CFR=Coronary Flow Reserve ; EQ-5D-5L=European Quality-of-Life-5 Dimension-5 Level questionnaire; PCI=Percutaneous Coronary Intervention; PROM=Patient-Reported Outcome Measure; SAQ=Seattle Angina Questionnaire

**Table S12. Spearman correlation between corrected IMR (IMRc) and Patient Reported Outcome Measure scores 3 months post-PCI among patients with angina at baseline (CCS Class I and above)**

| Patient Reported Outcome Measure | IMRc | Correlation Coefficient | P value |
|---|---|---|---|
| SAQ Physical Limitation Score (SAQ7-PL) | Baseline | -0.02 | 0.82 |
| | Post-PCI | 0.02 | 0.70 |
| | Absolute Change | -0.02 | 0.85 |
| | Percentage Change | 0.04 | 0.64 |
| | | | |
| SAQ Angina Frequency Score (SAQ7-AF) | Baseline | -0.04 | 0.62 |
| | Post-PCI | -0.05 | 0.54 |
| | Absolute Change | -0.03 | 0.67 |
| | Percentage Change | -0.03 | 0.73 |
| | | | |
| SAQ Quality of Life Score (SAQ7-QL) | Baseline | -0.04 | 0.59 |
| | Post-PCI | -0.04 | 0.59 |
| | Absolute Change | 0.02 | 0.84 |
| | Percentage Change | 0.04 | 0.64 |
| | | | |
| SAQ Summary Score (SAQ7-SS) | Baseline | -0.03 | 0.68 |
| | Post-PCI | -0.04 | 0.56 |
| | Absolute Change | -0.01 | 0.88 |
| | Percentage Change | 0.02 | 0.78 |
| | | | |
| EQ-5D-5L UK Weighted Health Index | Baseline | -0.08 | 0.29 |
| | Post-PCI | -0.08 | 0.26 |
| | Absolute Change | -0.02 | 0.81 |
| | Percentage Change | 0.01 | 0.95 |

CCS=Canadian Cardiovascular Society; IMRc=Index of Microcirculatory Resistance corrected for epicardial stenosis (Yong's formula); EQ-5D-5L= European Quality-of-Life-5 Dimension-5 Level questionnaire; PCI=Percutaneous Coronary Intervention; SAQ=Seattle Angina Questionnaire.

**Table S13. PROM scores 3 months post-PCI stratified by tertiles of IMRc at baseline, post-PCI and percentage change among patients with angina at baseline**

| IMRc | | Low | | Intermediate | | High | | p value |
|---|---|---|---|---|---|---|---|---|
| | **Patient Reported Outcome** | **N** | **Score** | **N** | **Score** | **N** | **Score** | |
| Baseline | SAQ Physical Limitation Score | 53 | 83.73±25.32 | 47 | 78.37±26.28 | 50 | 76.83±29.66 | 0.40 |
| | SAQ Angina Frequency Score | 56 | 86.61±20.56 | 53 | 83.58±21.93 | 56 | 84.82±22.07 | 0.76 |
| | SAQ Quality of Life Score | 56 | 80.58±25.99 | 53 | 74.29±28.42 | 56 | 77.01±30.60 | 0.51 |
| | SAQ Summary Score | 56 | 83.59±20.09 | 53 | 79.12±22.84 | 56 | 79.42±25.14 | 0.51 |
| | EQ-5D-5L Health State (UK) | 56 | 0.84±0.24 | 53 | 0.79±0.23 | 56 | 0.78±0.27 | 0.39 |
| Post-PCI | SAQ Physical Limitation Score | 60 | 77.50±27.07 | 51 | 86.60±23.57 | 55 | 76.59±27.95 | 0.10 |
| | SAQ Angina Frequency Score | 63 | 85.08±21.01 | 60 | 88.67±18.91 | 60 | 83.83±22.33 | 0.42 |
| | SAQ Quality of Life Score | 63 | 76.98±27.42 | 60 | 80.83±27.47 | 60 | 74.79±30.14 | 0.50 |
| | SAQ Summary Score | 63 | 80.18±21.63 | 60 | 85.13±22.06 | 60 | 77.87±23.71 | 0.20 |
| | EQ-5D-5L Health State (UK) | 63 | 0.81±0.23 | 60 | 0.84±0.26 | 60 | 0.77±0.23 | 0.37 |
| Absolute Change | SAQ Physical Limitation Score | 47 | 79.61±27.68 | 50 | 80.67±25.22 | 46 | 78.17±28.75 | 0.90 |
| | SAQ Angina Frequency Score | 53 | 85.85±20.80 | 55 | 87.27±19.76 | 50 | 82.00±22.95 | 0.43 |

| | | | | | | | | |
|---|---|---|---|---|---|---|---|---|
| | SAQ Quality of Life Score | 53 | 76.65±29.83 | 55 | 79.32±26.38 | 50 | 76.50±28.86 | 0.85 |
| | SAQ Summary Score | 53 | 80.46±24.03 | 55 | 82.81±21.01 | 50 | 78.84±23.48 | 0.67 |
| | EQ-5D-5L Health State (UK) | 53 | 0.80±0.28 | 55 | 0.83±0.19 | 50 | 0.78±0.26 | 0.54 |
| Percentage Change | SAQ Physical Limitation Score | 47 | 77.84±29.61 | 48 | 82.29±24.04 | 48 | 78.39±27.57 | 0.68 |
| | SAQ Angina Frequency Score | 54 | 85.19±21.61 | 53 | 88.30±18.99 | 51 | 81.76±22.60 | 0.29 |
| | SAQ Quality of Life Score | 54 | 75.69±29.78 | 53 | 81.37±26.47 | 51 | 75.49±28.39 | 0.48 |
| | SAQ Summary Score | 54 | 79.50±24.77 | 53 | 84.34±20.74 | 51 | 78.39±22.52 | 0.36 |
| | EQ-5D-5L Health State (UK) | 54 | 0.79±0.28 | 53 | 0.84±0.19 | 51 | 0.78±0.25 | 0.46 |

EQ-5D-5L= European Quality-of-Life-5 Dimension-5 Level questionnaire; IMRc=Index of Microcirculatory Resistance corrected for epicardial stenosis (Yong's formula); PCI=Percutaneous Coronary Intervention; PROM=Patient-Reported Outcome Measure; SAQ=Seattle Angina Questionnaire
